# The complete mitochondrial genome of *Thryssa hamiltonii* (Engraulinae, Engranlidae, Clupeoidei) and phylogenetic studies of Clupeoidei

**DOI:** 10.1080/23802359.2018.1424584

**Published:** 2018-01-10

**Authors:** Hui Jiang, Zhenming Lü, Liqing Liu, Bingjian Liu, Li Gong

**Affiliations:** National Engineering Laboratory of Marine Germplasm Resources Exploration and Utilization, College of Marine Science and Technology, Zhejiang Ocean University, Zhoushan, People’s Republic of China

**Keywords:** *Thryssa hamiltonii*, mitogenome, phylogenetic relationship

## Abstract

The complete mitochondrial genome of *Thryssa hamiltonii* has been determined. The whole sequence was 16,894 bp in length and included 13 protein-coding genes, 22 transfer RNA genes, two ribosomal RNA genes and one control region (D-loop). The overall base composition is A 30.77%, C 27.97%, G 16.25%, T 25.01%, with a slightly A + T bias of 55.78%. With the exception of *ND6* and eight tRNA genes, all other mitochondrial genes are encoded on the heavy strand. Three tandem repeat sequences were observed in the control region. Phylogenetic tree was constructed based on 13 protein-coding genes sequences of 21 clupeoidei species, and the result showed that *T. hamiltonii* is most closely related to *T. dussumieri.* These mitogenome sequence data will be useful for population genetics and phylogenetic analysis of the Clupeoidei.

*Thryssa hamiltonii,* which belongs to Engraulinae, Engranlidae, Clupeoidei, is distributed in the western Indo-Pacific (PJP et al. 1988; J.S. 1994). Although there were some studies on its fishery biology (Qin et al. 2011) and population structure (AL-HASSA 1988), little information about its genetic characteristics is available. In order to find new DNA markers for the future research of population genetics and phylogenetics and taxology, we determined the complete mitogenome of *T. hamiltonii* (GenBank accession number MF668229) by PCR amplification and primer walking sequence method.

*Thryssa hamiltonii* was collected from the South China Sea (18°12'30”N 109°28'49”E) and stored in a refrigerator of −80 °C with accession number 20161015TH04. The specimen was identified based on the morphologic features and COI gene. Muscle tissues of individual specimens for molecular analysis were reserved in ethanol absolute. Whole genomic DNA was extracted by using the phenol-chloroform method (Barnett and Larson 2012). The quality of the genomic DNA was checked using 1% agarose gel. The universal primers (Ivanova et al. 2007) were designed from the conserved regions of the complete mitochondrial genome sequences of 26 Clupeiformes species from GenBank database.

The complete mitochondrial genome of *T. hamiltonii* was 16,894 bp in length, consisting of 13 protein-coding genes, 22 transfer RNA genes (tRNA), two ribosomal RNA genes (12S rRNA and 16S rRNA) and one control region (D-loop). Except *ND6* and eight tRNAs (Gln, Ala, Asn, Cys, Tyr, Ser, Glu, Pro), other genes were encoded on the heavy strand. The mitochondrial base composition is A 30.77%, C 27.97%, G 16.25%, T 25.01%, respectively. The A + T content (55.78%) is higher than G + C content, in common with other Clupeoidei mitogenomes (Bi and Chen 2011; Li et al. 2012; Qiao et al. 2012; Bo et al. 2013; Wang et al. 2015; Zhang et al. 2016). Twelve protein-coding genes start with ATG except *COX1* with GTG. For the stop codon, *ND6* ends with TAG, seven genes with TAA, *ND2*, *ND3*, *COX2*, *ND4* and *CYTB* with an incomplete TA or T. The 12 S rRNA (954 bp) is located between tRNA^Phe^ and tRNA^Val^ genes, and 16S rRNA (1690 bp) is located between tRNA^Val^ and tRNA^Leu^ genes. The control region (D-Loop) typically located between tRNA^Pro^ and tRNA^Phe^ genes, is 1246 bp in length. Three tandem repeat sequences were observed in the control region. The shortest motif was 39 bp with five repeats, and other two motifs were both 117 bp with two repeats. The symbolic structures of the control region are observed as in other fishes, such as the TAS-cTAS, central conserved sequence blocks (CSB-F, D, B, A), CSB 2-3, G-box (GTGGGGG), and a pyrimidine tract (Guo et al. 2003; Gong et al. 2015).

Phylogenetic tree (Figure 1) was constructed using the neighbour-joining (NJ) method based on the 13 protein-coding genes of 21 clupeoidei species. The result shows that *T. hamiltonii* is most closely related to *T. dussumieri*. We expect the present results will further facilitate for the study on the taxonomy, population genetic structure and phylogenetic relationships of Clupeoidei.

**Figure 1.**
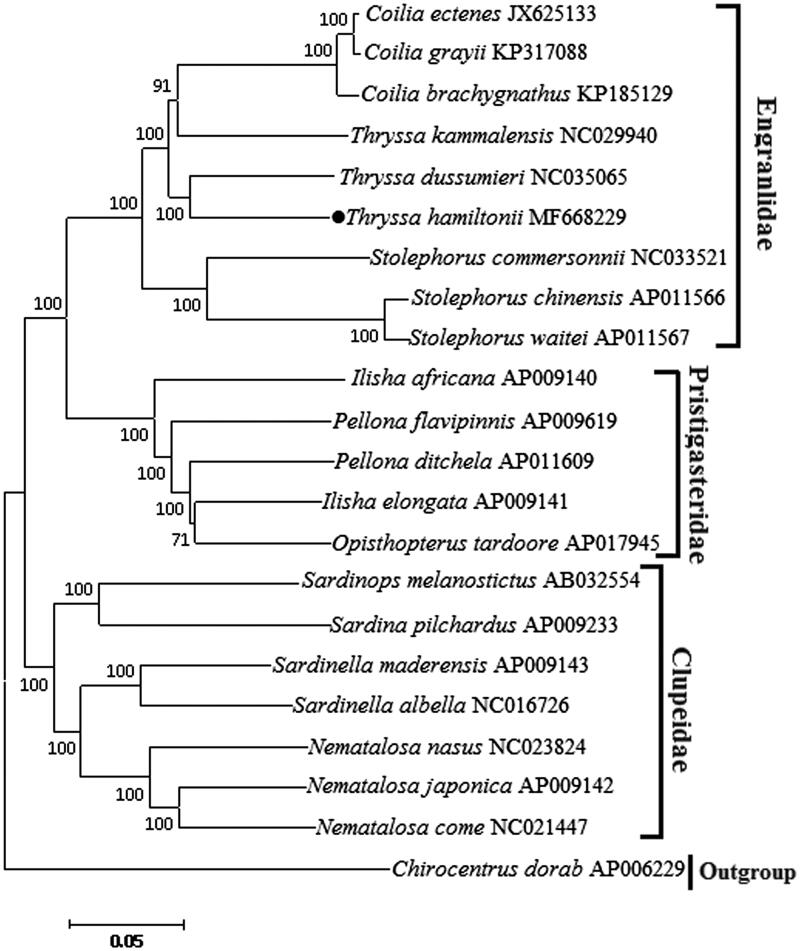
Neighbour-joining tree was constructed based on 13 protein-coding genes of 21 Clupeoidei complete mitogenome. The black dot indicated the species in this study. The number at each node is the bootstrap probability. The number before the species name is the GenBank accession number.
